# Claspin and Cancer: Where Are We Now?

**DOI:** 10.3390/ijms26188828

**Published:** 2025-09-10

**Authors:** Diana Azenha, Teresa C. Martins

**Affiliations:** 1 Faculdade de Farmácia da Universidade de Coimbra, Pólo das Ciências da Saúde, Azinhaga de Santa Comba, 3000-548 Coimbra, Portugal; 2 Innovative Therapies: Tumor Microenvironment and Targeted Therapies, Center for Neuroscience and Cell Biology, Faculdade de Medicina, University of Coimbra, Rua Larga, Pólo I, 1º andar, 3004-504 Coimbra, Portugal; 3 Molecular Pathology Laboratory, Portuguese Institute for Oncology at Coimbra Francisco Gentil, EPE, Av. Bissaya Barreto, 98, Apartado 2005, 3000-651 Coimbra, Portugal

**Keywords:** Claspin, cancer, replication stress, cancer therapy

## Abstract

Cancer cells arise from the cumulative acquisition of genetic and epigenetic alterations that affect vital cellular functions. Genomic instability results from deficiencies in protective mechanisms, such as cell cycle checkpoints, DNA replication control, or DNA repair. Claspin integrates a group of crucial proteins that maintain genome integrity. It participates in key cellular events such as DNA damage checkpoint activation, DNA replication, replication stress responses, DNA repair, epigenetic memory, and apoptosis. Given its crucial functions, a role for Claspin in cancer is not a surprise. Indeed, there is a considerable body of evidence linking Claspin deregulation with cancer. For instance, over-expression of Claspin and Tim promoted the survival of cancer cells by enabling adaptation to oncogene-induced replication stress. In addition, Claspin gene (*CLSPN*) mutations that affect checkpoint regulation have been identified in cancer patients, suggesting that they may contribute to cancer development. Changes in Claspin expression levels may be used as a prognostic marker in several types of cancer. Finally, several therapy-resistance signaling pathways seem to converge onto Claspin’s stabilization, turning Claspin into an attractive target for chemo- and radio-sensitization. In this review, we will focus on the role of Claspin in cancer and ways in which Claspin can be exploited in cancer therapy.

## 1. Introduction

Cancer is one of the most common causes of mortality worldwide. Cancer is generally characterized by specific hallmarks that drive cancer development and progression, including sustained and uncontrolled proliferation, evasion of growth suppression, cell death resistance, angiogenesis, invasion and metastasis, replicative immortality, deregulated cellular metabolism, subversion of inflammatory cells, and immune evasion [1,2]. One of the enabling features of cancer is genome instability, which constitutes a source of genetic variability that can foster malignant transformation or provide adaptive and survival advantages to cancer cells, constituting the major driving force for tumor initiation and progression. To maintain genomic integrity and prevent DNA damage, cells rely on checkpoints that function as barriers that halt cell cycle progression at critical points, whenever damage is detected, to provide the cell time to activate repair pathways and resume the cell cycle, or to activate cell death [3]. Most cancers present detrimental mutations in genes involved in DNA monitoring and repair, as well as cell cycle regulation [1,2]. Through the coordinated regulation of gene expression and protein localization and function, the cell ensures smooth cellular growth and proliferation. Deregulation of the cell cycle is central to oncogenesis, with compromise of checkpoints and unscheduled proliferation, features associated with replication stress (RS) and genome instability [4]. One key protein involved in checkpoint activation is Claspin, which acts as a scaffold protein that mediates the phosphorylation of the effector kinase Chk1 by ATR [5]. Claspin also has a role in the maintenance of genome integrity, checkpoint activation, and DNA repair [6], or apoptosis induction when damage is unrepairable [7,8]. In addition, Claspin participates in replication monitoring, being a component of the replisome and the replication fork protection complex (FPC) [9,10]. Recently, Claspin was shown to have a role in epigenetic memory, a process that ensures commitment of cells into different lineages [11]. Claspin is therefore a multifunctional protein that participates in essential processes involved in the maintenance of cellular homeostasis, which is frequently altered in cancer. Current evidence suggests that Claspin may have a significant role in carcinogenesis. Claspin inactivation seems to be an essential event in oncogenesis, a feature compatible with a role as a tumor suppressor. However, other studies have shown a direct correlation between Claspin levels in tumors and worse prognosis, as well as cancer progression, features that are suggestive of a role as an oncogene. Therefore, Claspin may have a more complex role in cancer than previously anticipated, which may be dependent on the cellular context, the mutational landscape of tumors, and the stage of oncogenesis. In addition, Claspin seems to play a role in the tumors’ response to chemo- and radiation therapy, acting as a pivot onto which different pathways converge to promote resistance of tumor cells to therapy. As such, Claspin-targeted therapies may have great potential in the context of radio- and chemo-sensitization. In this review, we will try to integrate the currently available data on the role of Claspin in cancer and discuss how Claspin can be exploited in patients’ follow-up and cancer treatment.

## 2. Claspin

Claspin was first identified during cell cycle checkpoint regulation experiments as a crucial protein for *Xenopus* Chk1 (xChk1) phosphorylation and ATR-Chk1 checkpoint activation following replication blockade and/or ultraviolet (UV) exposure [5]. Later studies led to the definition of a molecular circuitry in which Claspin acts as an adaptor or scaffold protein that facilitates the activation of Chk1 by ATR in the context of a complex composed of multiple proteins that cluster on single-stranded DNA (ssDNA), namely at stalled replication forks and sites of DNA damage [5,12,13]. Claspin was shown to be a vital protein, as Claspin (*CLSPN^−^*^/−^) knockout (KO) mice are embryonically lethal [14], as observed for other proteins, such as BRCA1 [15,16] and Chk1 [17,18].

**Figure 1:**
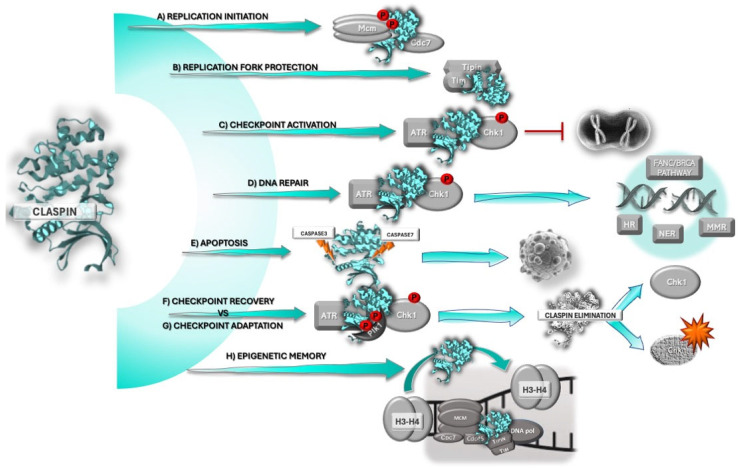
Claspin participates in several cellular mechanisms that are usually altered in cancer. Claspin is a component of the replisome, which regulates replication and origin firing (A), and of the replication fork protection complex (FPC), being responsible for the stabilization of replication forks (B). Claspin is an essential scaffold protein in Chk1 phosphorylation by ATR during checkpoint activation and cell cycle arrest (C). In addition, Claspin bridges checkpoint activation and different DNA repair pathways (homologous recombination, HR; nucleotide excision repair, NER; mismatch repair, MMR; and FANC/BRCA pathway) (D). If DNA damage is too extensive and cannot be repaired, Claspin is cleaved by caspases-3 and -7, and is degraded in the proteasome, which drives the cell into apoptosis (E). After DNA damage repair, Plk1 phosphorylates Claspin, promoting its degradation by the ubiquitin–proteasome pathway. Claspin elimination allows for checkpoint recovery and cell entry into mitosis (F), or checkpoint adaptation (G), a process in which cells undergo mitosis despite sustained DNA damage. Claspin also plays an important role in epigenetic memory (H).

Claspin participates in several key cellular functions that are usually altered in cancer (Figure 1). During the S-phase (Figure 1A), Claspin couples DNA polymerization with helicase activity and monitors ongoing replication to ensure precise genome duplication [19]. Claspin is a component of the replisome [20,21], which forms at replication origins, regulating origin firing [9], and is fundamental to maintaining normal replication rates [22]. It is also part of the replication fork protection complex (FPC), thereby participating in the stabilization of replication forks (Figure 1B). Following genotoxic stress [10] (Figure 1C), Claspin brings ATR and Chk1 together for Chk1 phosphorylation by ATR, thereby triggering the Chk1-mediated cell cycle checkpoint [5,13], which promotes cell cycle arrest, so that cells have time to engage in adequate localized responses, such as DNA damage repair. Claspin seems to lie at the heart of a switch that, upon DNA damage, drives the cell either into cycle arrest and DNA repair [23,24,25] (Figure 1D), or apoptosis, when damage is too extensive or unrepairable (Figure 1E) [7,8]. When DNA damage is successfully repaired, Claspin is degraded for checkpoint termination or recovery (Figure 1F), and the cell resumes the cell cycle [26,27,28]. However, sometimes the cell may proceed to mitosis despite the presence of DNA damage, in a process called “adaptation”, which also involves Claspin degradation (Figure 1G). Another enabling feature of cancer, likely involved in tumor cell plasticity and dedifferentiation, is epigenetic reprogramming [2]. Recently, Claspin was shown to have a role in epigenetic memory (Figure 1H), namely in the recycling of histones H3–H4 on the leading strand of DNA [11].

Due to the importance of its functions in cell homeostasis, Claspin expression must be tightly regulated. Similar to other crucial proteins, such as p53, Claspin is an unstable protein, whose expression is regulated at the transcriptional, post-transcriptional, and post-translational levels. Claspin expression oscillates throughout the cell cycle, being nearly absent in G0/G1, peaking at S/G2, and declining abruptly at the onset of mitosis [5,13,26,27,28,29]. The main process of Claspin expression control is degradation through the ubiquitin–proteasome pathway, through the coordinated action of multiple ubiquitin ligases (e.g., APC/Cdh1 and SCFβTrCP) and deubiquitinases (DUBs) (e.g., USP9X, USP29, USP7, USP28, USP20) [30,31,32,33]. Tristetraprolin (TTP), an mRNA-interacting protein, was recently shown to stabilize Claspin mRNA, post-transcriptionally, by binding to its 3′UTR, and consequently maintain Claspin expression for replication fork progression and appropriate replication checkpoint activation during the RS response [34].

### 2.1. Claspin and the DNA Damage Response

The integrity of the cell’s genetic information is constantly challenged by exogenous and endogenous threats. To ensure that genetic information is faithfully transmitted during cell division, cells have developed highly complex defense mechanisms capable of detecting and dealing with a plethora of DNA lesions, which are collectively known as the DNA damage response (DDR). The DDR is triggered whenever a DNA lesion is detected by sensor proteins. The danger signal is then transmitted to effector proteins by transducer proteins through the action of mediator or adaptor proteins that function as bridges and/or scaffolds that promote protein–protein interactions and proper checkpoint activation. Effector proteins then act on specific substrates, either inducing cell cycle arrest, providing the cell time to activate downstream repair actions and resume the cell cycle, or, if the damage is irreparable, cell death, to preserve genome integrity [5]. Depending on the type of lesion, DNA damage checkpoint activation takes place mainly through the ATR–Chk1 or ATM–Chk2 pathways [35,36].

The ATR–Chk1 pathway is activated predominantly in response to single-strand breaks (SSBs) and DNA lesions arising from RS, although it can also be activated by double-strand breaks (DSBs). RPA has a high affinity for ssDNA stretches arising from genotoxic stress or RS, stabilizing them and preventing further breakage. The RPA-ssDNA conjugate constitutes the trigger for the DDR response and ensures the recruitment of ATR-activating proteins to the site of damage [36,37]. ATR is recruited to RPA-ssDNA by ATRIP and is activated by TOPBP1or ETAA1 [37]. Once activated, ATR phosphorylates and activates the downstream effector kinase Chk1, a process that requires Claspin, which acts as an adaptor (Figure 1C) [36,38]. Following its activation, Chk1 phosphorylates and suppresses the cell cycle activator Cdc25C phosphatase, and phosphorylates the cell cycle inhibitor Wee1 kinase, promoting its activity, which results in cell cycle arrest [12,37]. During RS, Chk1 is responsible for several processes that prevent DNA synthesis until replication can resume, such as stabilization of stalled replication forks and suppression of origin firing [12,37,39]. As already mentioned, DSBs may also activate the ATR-Chk1 checkpoint [40]. In the presence of DSBs, Claspin associates with BRCA1, forming a complex that promotes checkpoint activation through Chk1 [41,42]. Claspin’s role in DSB damage response is dependent on the phosphorylation of specific residues that are distinct from those that are phosphorylated during checkpoint activation in response to DNA replication blocks [42]. Due to its functions, Chk1 activation must be tightly regulated [12]. Chk1 activation can be enhanced by promoting Claspin stability. Over the years, a growing number of DUBs have been identified that function as regulators of Claspin stability. Overall, regulation of Claspin ubiquitination allows the fine-tuning of Claspin availability and stability, which, in turn, regulates Chk1 activation and the DDR itself (for more details, see [6]).

Claspin also seems to play a role in bridging the ATR-Chk1 cell cycle checkpoint with DNA damage repair pathways (Figure 1D) (for a detailed description, see [43]). BRCA1 is a key protein in homologous recombination (HR). BRCA1 mediates Chk1 activation and promotes its interaction with and phosphorylation of two HR pivotal proteins: RAD51 and BRCA2 [44,45]. Claspin directly interacts with BRCA1 and regulates its phosphorylation, contributing to Chk1 activation following genotoxic stress [41]. BRCA1 can acquire E3 ubiquitin ligase activity through heterodimerization with BARD1 and target Claspin. In response to camptothecin, a genotoxic agent, Claspin complexes with Chk1 and BRCA1 on chromatin, triggering the phosphorylation of Chk1 by ATR. Claspin ubiquitination by BRCA1/BARD1 seems to promote Claspin accumulation and permanence on chromatin by a mechanism yet to be clarified, but possibly associated with the regulation of Claspin turnover. This favors Chk1 activation and triggers HR. Inhibition of BRCA1 ubiquitinase function decreased chromatin-bound Claspin, impairing HR [46]. Claspin may also have a role in mismatch repair (MMR). Co-immunoprecipitation experiments using nuclear extracts of a proficient MMR cell line revealed the interaction of Claspin with MSH6 and PMS2, suggesting that Claspin may interact with both MutSα and MutLα MMR complexes, either indirectly or through post-translational modifications [24]. Claspin also interacts with DNA damage-sensing proteins of the nucleotide excision repair (NER) pathway, namely DDB1, DDB2, and XPC. Silencing experiments demonstrated that Claspin impacts DDB2 in two ways: Claspin seems to be required for DDB2 turnover by enabling its ubiquitination and degradation, and for DDB2 recruitment and localization at damaged chromatin foci, which is essential for DDB2 damage recognition [25]. Finally, Claspin was implicated in the FANC/BRCA pathway. It was shown that Claspin is required for efficient FANCD2 mono-ubiquitination and assembly in subnuclear foci in response to DNA damage [23]. These data strongly suggest that Claspin acts as a bridge between cell cycle checkpoints and DNA repair pathways. However, its precise role in these mechanisms remains to be fully understood.

When the cell is unable to repair DNA damage, or the cell cycle arrest is too prolonged, the checkpoint response must be terminated, and the cell induced to die by apoptosis. Claspin seems to act as a brake against apoptosis in cells with DNA damage or replication blocks. However, when DNA damage is too extensive, this brake must be relieved, and the cell induced to die. Brake release is dependent on Claspin degradation (Figure 1E) through the action of caspase-7 (and caspase-3), and the proteasome [7,8]. Caspase-7 cleaves Claspin in the nucleus, promoting its dissociation from Chk1 and generating a fragment with a dominant-negative action on the checkpoint response and cell cycle arrest [7]. Progression to full-blown apoptosis requires full Claspin degradation by both the proteasome and effector caspases [8]. Proteasomal degradation of Claspin may amplify the effect of the dominant-negative fragments produced by caspase-7. The existence of two degradation pathways may constitute a backup safety mechanism that guarantees Claspin inactivation and apoptosis induction when DNA damage is too extensive, so that genome integrity is preserved [8]. When DNA damage is repaired, the checkpoint response must be deactivated for the cell to resume the cell cycle. This process is called “checkpoint recovery” or “checkpoint termination”, and requires Claspin destabilization and degradation so that Chk1 activation is blocked (Figure 1F). Claspin is degraded through the ubiquitin–proteasome pathway, upon the action of SCFβTrCP ubiquitin ligase complex, early in mitosis [26,28], after Claspin phosphorylation by Plk1 [26,27]. Nevertheless, some cells may still resume the cell cycle in spite of DNA damage, in a process termed “checkpoint adaptation” (Figure 1G), which seems to take place after a prolonged interphase block, as demonstrated by the detection of persistent stalled replication forks in mitosis [47]. In *Xenopus*, checkpoint adaptation involves the action of Plx1 (Plk1 homolog), which associates with and phosphorylates Claspin after xATR-mediated phosphorylation. The phosphorylation of Claspin by xATR and Plx1 promotes Claspin dissociation from chromatin, xChk1 inactivation, and cell entry into mitosis. Syljuåsen and colleagues [48] showed that human checkpoint adaptation also required Plk1-dependent Chk1 inactivation. However, the role of Claspin in the process remains to be elucidated.

### 2.2. Claspin’s Role in DNA Replication and Replication Stress

Claspin’s role in DNA replication is well established, although not fully understood (for more details, refer to [43]). It is clear, however, that its function in replication is independent of its role in checkpoint signaling [49]. As mentioned above, Claspin is an integral component of the replisome [20,21], which is loaded onto replication origins, and directly monitors DNA synthesis during the S-phase of the cell cycle [50,51,52]. Human Claspin ring-shaped protein binds DNA and has a high affinity to branched DNA structures that arise at stalled replication forks, sites of DNA damage, and unwound DNA during replication [5,51,52]. Claspin interacts with several components of the replication machinery, namely Tim, MCM proteins, DNA polymerases (DNApol) α, δ, ε, Cdc7, Cdc45, and OZF [50,51,53,54,55]. Claspin is required for normal replication rates [22], as well as for the maintenance of replication fork stability [52]. Claspin is also a crucial regulator of Cdc7 recruitment to replication origins and the pre-replication complex in normal cells [55]. Cdc7 phosphorylates the MCM complex in the initiation of replication, promoting its association with other proteins and the formation of an active replication complex [55]. In this way, Claspin participates in the control of DNA replication and the prevention of DNA re-replication.

Claspin is a component of the replication fork protection complex (FPC), along with Tim, Tipin, and And-1 (Figure 1B). This complex participates in the maintenance and monitoring of replication fork stability, cohesion of sister chromatids, and inhibition of myotonic dystrophy protein kinase (DMPK) gene (CTG)(n)·(CAG)(n) repeat instability [10,20,51,56,57]. Claspin and Tim also have a role in replication protection following DNA damage (e.g., ssDNA gaps and DNA breaks) by promoting PCNA monoubiquitination. This facilitates PCNA interaction with translesion DNApol, which allows for replication to proceed in the presence of DNA damage [58]. Claspin also protects genome integrity through common fragile site monitoring [59]. Common fragile sites are large, highly unstable and recombinogenic regions of the genome that are difficult to replicate and prone to breaking under stress, thereby contributing to genomic instability and cancer development [60]. The ATR–Claspin–Chk1 pathway is responsible for the faithful replication of these regions, as well as for the stabilization of common fragile sites, by steadying stalled replication forks and preventing fork collapse, thus contributing to genome integrity [61,62,63]. It has been shown that Claspin is readily recruited to chromatin, as soon as replication forks slow down [64], and that Claspin silencing results in genomic abnormalities in the form of specific fragile site expression [59].

DNA replication must be coordinated with the duplication of chromatin structures to guarantee faithful transmission of specialized states of chromatin onto daughter cells [11]. This process is pivotal for the maintenance of cell identity during development, and to prevent changes in cell function that may occur in aging and malignant transformation. The inheritance of parent cell histones across the replication fork is believed to mediate epigenetic memory. The replisome seems to be responsible for the transmission of the histone code. It was recently shown that Claspin is required for recycling of parental H3–H4 histones to the leading strand (Figure 1H), whereas the yeast homologue, Mrc1, has a dual function in histone recycling to both the leading and the lagging strand. Mrc1 seems to operate as a pivotal coordinator of symmetric parental histone transmission, in part by intra-replisome co-chaperoning with Mcm2. Of note, although it is still not clear whether Claspin also has a dual function in histone recycling to both the leading and lagging strands, there is a similar interaction with Mcm2. Malfunctioning of this mechanism may result in loss of parental histones and loss of epigenetic memory [11], a process that may contribute to cancer cell dedifferentiation.

RS occurs whenever replication is blocked or perturbed. RS sources include unrepaired DNA lesions, repeated sequences that may form secondary structures (e.g., hairpins, triplets), and replication and transcription machinery collisions, among others. RS usually results in ssDNA stretches that are coated with RPA, triggering an RS response led by the ATR–Claspin–Chk1 pathway [65]. Claspin acts as the interface between stalled replication forks and the activation of the ATR-Chk1-mediated cell cycle checkpoint, which is responsible for several local responses, such as slower replication fork progression, replication fork stabilization, and inhibition of origin firing, until damage is resolved [38]. The efficient activation of the ATR-Claspin-Chk1-mediated replication checkpoint in response to RS requires Cdc7 and CK1γ1 [66,67,68]. Normal cells seem to rely predominantly on CK1γ1, whereas cancer cells mostly rely on Cdc7 [68]. Cdc7-dependent phosphorylation of Claspin promotes its accumulation on chromatin [66] and effective Chk1 activation [66,67,68]. Phosphorylation of Claspin by Cdc7 is essential for successful checkpoint signaling, but it is no longer required once the checkpoint signaling is activated [67].

RS may also arise when the timing of replication, which is usually defined in early G1, is altered due to oncogene activation, which induces firing of replication origins in genes with high transcription rates. Claspin is also involved in an evolutionary conserved genome defense mechanism mediated by p38 that operates during the S-phase [69], particularly during osmostress and transcription–replication conflicts, with consequent formation of R-loops. Claspin over-expression can revert oncogene-induced RS independently of ATR signaling. The mammalian cell response to osmostress seems to be highly complex and involve the activation of Chk2, which can also phosphorylate Claspin [69]. Activation of the p38–Claspin pathway prevents the occurrence of R-loops, decreasing the overall burden of DNA damage and delaying the cell cycle [69]. Therefore, the p38–Claspin pathway appears to constitute an additional layer of protection against genomic instability, which is essential for cell survival and viability, and prevention of carcinogenesis.

## 3. Claspin and Cancer

As Claspin has a role in several processes involved in the maintenance of genome integrity and the promotion of successful and uneventful cell division, it is plausible that it may have a role in cancer development. In 2017 and 2019 [6,43], we published two seminal papers discussing several ways Claspin could be involved in cancer. Since then, many data have been produced supporting our thesis that have brought about the discussion of whether Claspin acts as an oncogene or a tumor suppressor. Several studies have shown that Claspin inactivation is an essential step for the malignant transformation of host cells by oncogenic viruses [70,71,72], such as high-risk human papillomaviruses (HR-HPV). HR-HPV malignant transformation of the cervix is mediated by two major oncoproteins, E6 and E7, that act on different elements of the host cell cycle machinery, promoting their deregulation to establish a favorable replication environment for HPV. The main target of the E7 oncoprotein is pRb, a tumor suppressor. pRb binds to the E2F family of transcription factors, blocking S-phase entry [73]. HPV E7 promotes pRb degradation, E2F release, S-phase gene transcription, and uncontrolled cell proliferation [74]. This sustained and uncontrolled proliferation activates a checkpoint response, mediated by p53, that is counteracted by HR-HPV E6 oncoprotein. E6 destabilizes p53, a key tumor suppressor and transcription factor with anti-proliferative properties, by mediating its degradation via the ubiquitin proteolytic pathway [75]. Spardy and colleagues [71] have shown that HPV-16 E7-expressing cells are able to circumvent DNA surveillance mechanisms and enter mitosis despite the presence of DNA damage through enhanced Claspin proteolytic turnover and checkpoint adaptation. Since Claspin is essential to maintain DNA damage checkpoint activation, its degradation promotes a permissive environment, allowing cells with DNA damage to “escape” surveillance mechanisms and enter mitosis [71]. This creates a S-phase-like environment ideal for viral genome replication, but that also favors genomic instability and cervical cancer development. Similar to p53 and pRb, Claspin constitutes a target in HR-HPV infection. Therefore, Claspin may constitute an additional link between HR-HPV infection and malignant transformation, suggesting a role for Claspin in carcinogenesis as a tumor suppressor.

Claspin was also implicated in Epstein-Barr Virus (EBV) infection, which is associated with B-cell lymphoproliferative diseases or lymphomas. Like HPV, the success of infection depends on bypassing checkpoint responses. EBV mobilizes host STAT3, a transcription activator implicated in the induction of proliferative and anti-apoptotic genes, to relax the ongoing checkpoint response activated in B cells by infection [70]. STAT3 then promotes Claspin degradation by caspase-7. The consequent ATR-Chk1 checkpoint relaxation facilitates EBV infection by promoting host cell proliferation, despite intra-S-phase checkpoint activation [70]. In turn, the Hepatitis B virus (HBV) oncoprotein pX (HBV pX), implicated in hepatocarcinogenesis, induces G2-phase Plk1 expression in immortalized pX-expressing hepatocytes [72]. Plk1 phosphorylates Claspin, targeting Claspin for proteasomal degradation [26]. This leads to the resumption of checkpoint activation and cell entry into mitosis. HBV pX promotes DNA damage, and this premature checkpoint recovery or checkpoint adaptation allows for persistent HBV pX-induced DNA damage and host cell polyploidy, which promotes hepatocyte oncogenic transformation [72]. In general, Claspin degradation appears to be a key event in most oncovirus-mediated infections that alleviates the host cell ATR–Chk1 checkpoint pathway, allowing the escape of infected cells from surveillance mechanisms and their entry into mitosis despite the presence of DNA damage.

Together, these data suggest that Claspin inactivation is an important event in carcinogenesis, and that Claspin may act as a tumor suppressor. Indeed, a Claspin-dependent, but ATM/ATR-independent, checkpoint response was shown to be lost during colorectal carcinogenesis [76]. In addition, a subgroup of patients with B-cell lymphoma with worse prognosis was identified, which was characterized by loss of SLF2. SLF2-deficiency leads to loss of components of the DDR machinery, including Claspin, a feature that impairs Chk1 activation and checkpoint responses [77]. It has also been shown that USP20 depletion was associated with increased chromosomal aberrations, malignant transformation, and tumor growth, and that the malignant phenotype could be significantly suppressed by ectopic expression of Claspin [40,41]. In addition, in gastric cancer, a correlation between Claspin expression and USP20 expression was observed, with low levels of these two proteins being associated with reduced overall survival. In contrast, high Claspin and USP20 expression were correlated with enhanced overall survival, pointing to a protective role for Claspin and USP20 in gastric cancer [78]. In turn, Bold and colleagues [79] found that patients with increased levels of Claspin mRNA presented increased survival probability after treatment for head and neck squamous cell carcinoma. Analysis of Claspin expression datasets revealed that low levels of *CLSPN* transcripts were significantly associated with a poor prognosis in several types of cancer, namely breast, stomach, head and neck, rectal, and gastric cancer [14,80].

*CLSPN* genetic variants are rather common in cancer [81,82,83,84,85,86] and may have implications on Claspin’s function and availability. However, these effects are often difficult to assess in a biological context. Several genetic alterations in breast and glioma tumor samples were identified [81,83], including two Claspin germline mutations and co-segregation of three polymorphisms in patients with breast cancer, which led to loss of Claspin expression in tumor cells [84], and in familial breast, breast-ovarian, and pancreatic cancer [82,85]. Although prediction software tools attributed functional significance to some of these variants, generally, no clear association with cancer has been established [82,85]. Nonetheless, one study showed that the *CLSPN* Ile783Ser variant impaired the ability of Claspin to mediate Chk1 phosphorylation following DNA damage, and to rescue sensitivity to RS in *CLSPN*-depleted cells [86]. *CLSPN* Ile783Ser missense mutation seems to behave as an attenuated allele that may influence tumorigenesis. We have also described a potentially highly pathogenic variant in *CLSPN* exon 8, c.1574A>G (p.Asn525Ser) [81]. This variant is associated with increased exon 8 skipping and may generate a premature stop codon at position 336 (p.Ser336Thrfs*13), leading to the production of a truncated form of Claspin. Functional assays showed that c.1574 A>G resulted in the partial loss (p.Ser336Thrfs*13) or decreased (p.Asn525Ser) Claspin expression [81]. This variant is located in a Claspin domain that is implicated in chromatin binding and Chk1 phosphorylation and can therefore impact checkpoint activation [51,87]. We have observed that the *CLSPN* exon 8 c.1574A>G variant was associated with reduced Chk1 phosphorylation, and hence, with decreased Chk1 activation. The p.Ser336Thrfs*13 transcript was totally unable to mediate Chk1 phosphorylation [81]. *CLSPN* truncating mutations have been detected in cancer samples in other studies. A somatic truncating mutation in *CLSPN*, p.Arg1040Ter (NP_071394.5), corresponding to the cDNA change c.3118C>T (NM_022111.5), was identified in a study that aimed at identifying novel candidate cancer genes that could account for early onset (<40 years) colorectal cancer [88]. This variant partially overlaps a domain that regulates origin firing and Claspin DNA binding [89]. However, the functional impact of this truncated form of Claspin was not assessed experimentally. Several reported genetic variants of *CLSPN* affect Serine/Proline (SP) sites, particularly prolines, altering p38 phosphorylation sites specifically. As such, these mutations may block the p38/Claspin pathway described above, which protects genome integrity [69]. Recently, *CLSPN* polymorphisms were shown to contribute to oral cancer progression, namely cell undifferentiation [90], which may be due to Claspin’s function in the transmission of the histone code into daughter cells [11]. In sum, Claspin inactivation seems to facilitate malignant transformation, a feature consistent with a role for Claspin as a tumor suppressor.

Reduced Claspin expression seems to promote initiation of tumorigenesis through deficient oncogene-induced DDR and increased genomic instability [12,85]. Heterozygous *CLSPN* knockout (KO) mice cells have reduced Claspin protein levels, while the other pathway partners (ATR and Chk1) are unaffected. It has been shown that, due to the low levels of Claspin and consequent reduced Chk1 activation, these cells were more resistant to the effects of Chk1 inhibitors than *CLSPN* wild-type (wt) cells. In addition, aged heterozygous knockout *CLSPN* mice were more susceptible to tumor development than *CLSPN* wt aged-matched littermates [12,80]. Heterozygous *CLSPN* KO mice were also more prone to develop acute liver injury induced by N-nitrosodiethylamine (DEN), a DNA alkylating agent that causes DNA and hepatocyte damage, as well as non-alcoholic fatty liver disease and hepatocellular carcinoma [12,80]. Hunter and colleagues [80] proposed that, in the early stages of carcinogenesis, *CLSPN* is a nuclear factor-kappa B (NF-kB) target gene that helps to prevent or reduce genome instability through its role in Chk1 activation, and therefore acts as a barrier in cancer development that results from RS. G2 checkpoint activation and oncogene-induced senescence are frequently found in pre-cancerous lesions but not in advanced tumors, suggesting that senescence may constitute an anti-cancer barrier. It has been shown that it is the duration of the Chk1-dependent G2 checkpoint that determines the entry of cells into senescence, and that this decision is dependent on Claspin expression and stabilization [91]. TRIM21 is an E3-ubiquitin ligase that is increased in many cancers. It has been shown that over-expression of TRIM21 may contribute to carcinogenesis through targeting of Claspin to degradation. Furthermore, TRIM21-mediated ubiquitination counteracts a ubiquitination event that is required for Claspin association with Tipin and consequent chromatin loading. Therefore, over-expression of TRIM21, through Claspin ubiquitination and degradation, compromises checkpoint responses (as it prevents Chk1 activation) and promotes replication fork instability (as it inhibits the formation of the FPC and the replisome), thus contributing to oncogenesis [92]. In addition, Claspin inactivation may facilitate cancer dedifferentiation, as Claspin is required for transmission of the histone code during cell division and for epigenetic memory [11]. Indeed, cellular differentiation, which is associated with impaired cell division, constitutes a major barrier against cancer [2]. Therefore, unlocking the phenotypic plasticity of cells, namely through loss of epigenetic memory, allows cells to escape from a state of terminal differentiation, which is crucial for carcinogenesis. All these studies support a role for Claspin as a tumor suppressor.

Nevertheless, there is also data that argues against a protective role for Claspin in cancer. Increased Claspin expression has been detected in a variety of cancer samples and cell lines and was associated with reduced patient survival. For instance, Benevolo and colleagues [93] suggested that high Claspin expression could be a biomarker for high-grade lesions of the uterine cervix associated with HPV infection. Claspin over-expression (along with that of Tim and Chk1) was associated with reduced disease-free survival in low-grade lung cancer [94]. Claspin expression was also upregulated in renal cell carcinoma and urothelial cancer and directly correlated with tumor grade and stage, nuclear grade, and vascular invasion, and was associated with worse prognosis [95,96]. Additionally, Claspin expression was correlated to that of other markers known to predict poor outcomes, such as CD44, EGFR, p53, and PD-L1, and was shown to be associated with cancer cell proliferation [95]. In gastric cancer, enhanced Claspin expression correlated with malignant histological parameters and with poor prognosis [97]. In low-grade glioma, high Claspin levels correlated with malignant histological characteristics and reduced overall survival, and were an independent risk factor for survival. Claspin expression was also associated with immune cell infiltration, which, in turn, predicted a worse prognosis. Based on these data, the authors suggested that *CLSPN* could act as an oncogene with strong prognosis and diagnostic value in low-grade glioma [98]. In line with these findings, Claspin over-expression was also detected in prostate cancer samples and cell lines and was associated with several prognostic markers related to poor outcome [99,100,101]. In vitro inhibition of Claspin expression decreased cell proliferation [99,100] and stimulated apoptosis [100], features consistent with the role of Claspin in cell cycle checkpoint signaling and cell-fate decisions, and restored the sensitivity to treatment of docetaxel-resistant prostate cancer cells [99]. In an integrated analysis of microRNA networks in hepatocellular carcinoma (HCC), *CLSPN* was found to be part of a set of genes regulated by miRNA-424, and low *CLSPN* expression was correlated with better overall patient survival, functioning as a prognostic marker for HCC [102]. *CLSPN* was also part of a set of genes proposed as a prognostic signature for HCC overall survival [103]. Interestingly, *CLSPN* presented a 6% mutation rate in HCC, which was significantly associated with vascular invasion [102].

Simian virus 40 (SV40) infection is associated with human primary brain tumors, malignant mesotheliomas, bone cancer, and non-Hodgkin’s lymphoma. The Large T antigen (LT) is a key mediator of the SV40 infection. The SV40 LT oncoprotein acts on the host cell machinery to create a permissive environment for viral genome replication. Although several oncogenic viruses act through DDR inactivation, others, such as SV40, take advantage of it. Indeed, SV40 LT activates both ATR- and ATM-mediated checkpoints, in part through interaction with Bub1, a mitotic spindle checkpoint kinase [104]. This interaction leads to p53 phosphorylation and stabilization through an ATR-dependent mechanism that may also involve Claspin, as LT and Claspin consistently co-immunoprecipitate in in vitro experiments [104]. Activation of DDR, through the ATR pathway, and ultimately p53, induces the transcription of the CDK inhibitor p21, promoting cell cycle arrest in the S-phase. This way, the cell stays in a replicative state, allowing viral replication and promoting cell cycle deregulation and genome instability [105].

Altogether, these data suggest that Claspin may instead act as an oncogene. So, where do these data leave us?

### 3.1. Claspin: Tumor Suppressor or Oncogene?

The importance of Claspin in cell homeostasis and its potential role in carcinogenesis is well established. The discussion now revolves around its role in cancer development: there are data that suggest a role as a tumor suppressor, as well as data pointing in the opposite direction—that of an oncogene. It is important to point out that the majority of the studies available so far are mostly based on the analysis of Claspin expression and are correlational studies. Little experimental research is available.

One of the ways the above-described contradictory findings may be explained is based on the cellular context. As mentioned, Claspin expression is correlated with different outcomes in tumors from distinct cell origins. Experimental data have also shown that *CLSPN* mutations may have different effects depending on the cellular context. For instance, we have observed that the impact of the *CLSPN* variant, c.−68C>T, on promoter activity was different depending on the cell line used (HEK and HeLa vs. U2OS cells) [81]. These different outcomes may result from the different molecular contexts of the distinct cell types used, namely, with regard to the availability of the transcription factors that may bind to this variant. In addition, detailed analysis of breast cancer data showed that low Claspin expression was associated with reduced survival, but only in patients who concomitantly presented with *TP53* mutations, whereas the opposite was found in patients with wt *TP53*. Similarly, in patients with HER2-positive breast tumors, low *CLSPN* mRNA levels correlated with reduced survival, with the opposite being observed in patients with HER2-negative tumors [80]. Therefore, the consequences of variations in Claspin expression may be more complex than currently described and depend on the other mutational events of the tumor. Claspin may generally act as a tumor suppressor, its mutation/inactivation having the potential to act as a driver mutation in cancer development, but its over-expression may also act as a secondary event that can correlate with a poor prognosis, or, in specific contexts, can amplify driver mutation events of given types of cancer (e.g., *TP53* mutation, HER2 over-expression). Regarding p53, the association is probably explained by the paramount role of both p53 and Claspin in cell cycle checkpoints. p53 plays a crucial role in G1/S, G2/M and replication checkpoints. While the G1/S checkpoint is completely dependent on p53, this is not the case for the G2/M and replication checkpoints. Upon sensing stress (e.g., DNA damage), p53 causes cell cycle arrest mainly by inducing the expression of the CDK inhibitor p21. p21 then binds to and inhibits the activity of cyclin-CDK 2/4 complexes, and, thus, functions as a regulator of cell cycle progression at G1 [106]. This is the first line of defense against genomic instability. However, p53 is mutated in more than half of human tumors, resulting in an inability to block cell cycle progression in the face of DNA damage, promoting error persistence, uncontrolled cell proliferation, and cancer development [107]. In this setting, if Claspin is also lost, or significantly reduced, the G2 and replication checkpoints will be further affected, since Claspin is essential for the activation of the ATR–Chk1 pathway. The loss of two hub proteins essential for checkpoint signaling would severely hinder the ability of the cell to block cell cycle progression in G1 and G2 in the face of DNA damage, fostering genome instability and favoring malignant transformation. Nevertheless, we believe this Achilles’ heel of tumors can be exploited in cancer therapy through the induction of further and extensive DNA damage, which cancer cells will not be able to repair and cope with, and will ultimately promote cancer cell death through mitotic catastrophe. Thus, another explanation for the observed discrepancies in the literature concerning Claspin’s role in cancer may originate from different patterns of association with other proteins that are mutated or whose expression is also altered in tumors. As an example, in early- to mid-stage non-small cell lung cancer, Claspin was only one of a set of five genes (POLQ, PLK1, RAD51, CDC6, and CLSPN) encoding proteins involved in the maintenance of genome stability that were found to be consistently upregulated and associated with poor survival [108].

**Figure 2:**
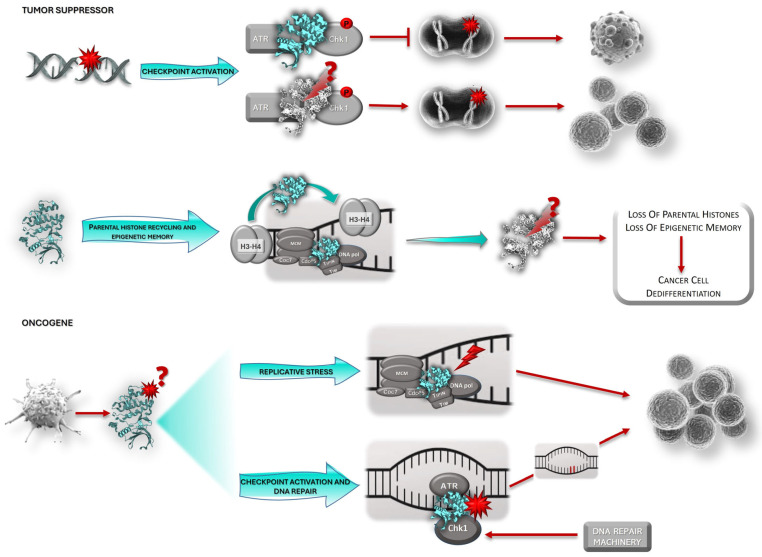
Claspin: tumor suppressor or oncogene? In early stages of oncogenesis, Claspin may act as a tumor suppressor (**top**), as its inactivation seems to be required for tumor initiation. Claspin loss of function prevents activation of the ATM-Chk1 checkpoint, an anti-cancer barrier that tumor cells must bypass to proliferate in spite of DNA damage. Claspin degradation allows for checkpoint adaptation and survival. In addition, as Claspin has a role in epigenetic memory, through recycling of parental H3–H4 histones to the leading strand, and maintenance of cell lineage features, its inactivation may contribute to tumor cell dedifferentiation and acquisition of stem cell features. However, in later stages of oncogenesis, Claspin may assume a role as an oncogene (**bottom**), or its functions may be exploited by cancer cells to survive and evolve. Tumor cells overcome oncogene-induced RS, by triggering an RS response mediated by the replisome and FPC, through over-expression of Claspin and Tim. In addition, Claspin’s role in checkpoint activation may be exploited by tumor cells to ensure their survival, namely when subjected to the selective pressure of RS, so that tumor cells can have time to attempt to repair RS-induced DNA damage, using the available repair pathways, and survive.

Another possible, and the most plausible, explanation regards the possibility of Claspin assuming different roles in different stages of oncogenesis (Figure 2) [89,97]. Claspin may act as a tumor suppressor at the initial phases of malignant transformation, protecting the cell from oncogenic pressure, but it is possible that in the later stages of carcinogenesis, it may acquire oncogenic properties, fostering cancer progression and survival. Tumors are evolving entities, and the acquisition of different malignant capabilities occurs over time. This process results in a multitude of cancer subtypes that reflect the many routes to cancer, which, in turn, are dependent on the ability of the tumor to bypass different defense barriers. Therefore, Claspin expression may be a mechanism exploited by cancer cells in later phases of tumor development to foster tumor survival, progression, and invasion, namely through checkpoint activation, which allows cells to delay the cell cycle and try to repair DNA damage, or allows tumor cells to deal with RS (Figure 2, bottom). For cancer cells, oncogene-induced RS is a double-edged sword because, although it promotes genomic instability (e.g., through replication fork collapse, alteration of chromatin assembly on newly replicated DNA strands, and reduction in DNA polymerase fidelity) and thus contributes to malignant transformation, it is also a burden for tumor cells, as it slows down replication and may trigger senescence. RS caused by oncogene activation drives constitutive activation of the DNA damage response (DDR) and checkpoint responses in pre-tumoral cells, allowing the bypass of anti-cancer barriers, including entry into senescence and apoptosis [94,109,110]. RS represents a source of genetic alterations that can confer adaptation advantages that will allow cancer cells to thrive and grow [1,100]. In addition, RS is also associated with malignant transformation through the induction of breaks at common fragile sites. Common fragile site instability is enhanced when the proteins involved in the protection of replication forks are mutated or when the balance between translesion DNA synthesis and DNA replication is perturbed [94]. In the initial phases of oncogenesis, Claspin may have to be silenced because Claspin activation in response to DNA damage would promote Chk1 activation, which in early oncogenesis, would inhibit cancer development through the prevention of genome instability (an enabling feature of cancer) in a dosage-dependent manner, and the accumulation of the genetic mutations required for cancer promotion (Figure 2, top) [1,110,111]. However, in the more advanced stages of tumorigenesis, this pathway can help to promote tumors’ addiction to checkpoint signaling, which is required for cell cycle arrest, the repair of RS- and therapy-induced DNA damage, and cancer cell survival (Figure 2, bottom). Indeed, Claspin’s role in checkpoint activation may be exploited by tumor cells to assure their survival, namely when subjected to the selective pressure of therapy or RS, so that tumor cells can have time to attempt to repair therapy- and RS-induced DNA damage, using the still available repair pathways, and survive. However, DNA is not always repaired using the most adequate pathways (which can be already inactivated in tumor cells) and, therefore, genome instability may increase, promoting tumor progression. This is clearly seen in EBV-induced B cell disorders. Early in infection and lymphomagenesis, STAT3 is activated, promoting activation of caspase-7, Claspin degradation, and the consequent suppression of Chk1 phosphorylation and checkpoint activation. However, at later timings, namely in immortalized cells, the role of STAT3 involves activation of the DDR, namely through phosphorylation of ATM and ATR, and their downstream effector kinases, Chk2 and Chk1, respectively [70]. It is also generally believed that tumor cells may adapt to oncogene-induced RS by modulating the intensity of the ATR–Claspin–Chk1 response [94,112,113], as ATR and Chk1 haploinsufficiencies enhance oncogene-induced tumor development [114,115]. In turn, Chk1 over-expression was shown to decrease oncogene-induced RS and promote tumor growth [116]. SV40 also exploits the DDR to replicate. Through interaction with Bub1, it activates both ATM- and ATR-mediated checkpoints, causing stabilization and activation of p53 in a process involving Claspin. Consequently, the cell cycle is arrested, allowing viral replication but also causing a deregulation of the cell cycle and promoting genome instability [104,105]. Understanding how cancer cells control this delicate balance represents a great challenge in cancer biology. Due to its central role in the ATR–Chk1 pathway and in replication fork stability, Claspin is pivotally placed for the fine-tuning of the cellular response to oncogene-induced RS. It was observed that the reduction in Claspin expression in colon carcinoma cell lines, to levels that did not promote checkpoint signaling, led to reduced fork speed, increased fork stalling, and accumulation of DNA damage. In turn, primary fibroblasts that escaped oncogene-induced senescence presented over-expression of Claspin and Tim, two components of the FPC, further supporting the notion of of cancer cells adapting to RS [94].

Thus, Claspin may assume different roles in different stages of oncogenesis. Indeed, Claspin expression may vary in different phases of tumor development. Benevolo and colleagues [93] assessed Claspin expression in cervical biopsy samples of increasing malignancy, including normal tissues, Cervical Intraepithelial Neoplasias (CIN) 1, 2, and 3, and squamous cell carcinomas, which represent different steps of malignant transformation. They found that Claspin expression increased with malignancy, being absent or scarce in normal tissue and significantly upregulated in squamous cell carcinomas. HR-HPV is the causative agent of cervical cancer. HPV-induced malignant transformation seems to require E7-mediated Claspin degradation, which compromises host cell checkpoint activation, thereby facilitating infection [71]. Therefore, it seems that, in a pre-malignant or early stage of carcinogenesis, HR-HPV abrogates Claspin expression to be able to replicate inside the host cell without triggering cell cycle checkpoints. However, as the process progresses to overt cancer, Claspin expression increases [93]. We believe that this increased expression can result from the HPV-induced RS or may be simply a reflection of the increased DNA replication promoted by the virus. Similar observations were made regarding other tumors, in which higher levels of Claspin expression were observed in tumors of higher grades than in lower-grade tumors or normal tissue samples (e.g., urothelium, prostate, renal cell carcinoma) [95,96,101], which is consistent with the presence of a higher number of proliferating cells in carcinoma than in normal tissues. All these data seem to support the notion that as tumors become out of control and their stemness features increase (e.g., high proliferation rate and RS), Claspin expression increases. This augmented expression makes sense as Claspin is used physiologically by cells to deal with RS. Therefore, it is conceivable that tumors exploit Claspin’s functions to deal with RS. Indeed, it has been shown that cells may adapt to oncogene-induced RS by spontaneous over-expression of Claspin and Tim, in a way independent of ATR (Figure 2, bottom). This seems to constitute a common strategy used by cancer cells to deal with RS without hyperactivating the ATR pathway and checkpoint response, which would be detrimental for tumor growth [94,110]. The mechanism through which RS tolerance is achieved is still under investigation. However, it seems that Tim is displaced from the replisome when cells are under RS in order to slow down the replication forks and prevent genome instability. In these circumstances, there will be an excess of free Claspin and Tim, both of which can promote reassembly of a functional FPC, which could then detect uncoupling of DNApol from DNA helicase activities, and transduce a signal to remodel and restart the stalled forks. This could be achieved through the promotion of PCNA ubiquitination, in an ATR-independent manner, a process required for translesion DNA replication. Therefore, over-expression of Claspin (and other FPC components) in cancer cells may constitute a reservoir of these proteins that allows the fast reassembly of functional FPCs that will promote fork restart and RS tolerance by a mechanism independent of the ATR-Chk1 checkpoint signaling pathway [94,110]. In addition, over-expression of POLQ, PLK1, RAD51, CDC6, and CLSPN was found to correlate with worse prognosis in non-small cell lung cancer [108]. The encoded proteins may modulate key aspects of the RS response, including replication fork recovery and licensing of dormant replication origins, backup origins that can be licensed in G1 but only in situations of RS [108]. In summary, although chronic RS may promote oncogenesis at its earliest stages, when the acquisition of mutations is crucial for tumor development, later it becomes a burden for tumor cells, as it ends up inhibiting proliferation, namely due to checkpoint activation. Therefore, cancer cells become reliant on an efficient RS response and Claspin for viability, and exploit fundamental Claspin function as an integral component of the FPC and the replisome as a way to survive. Therefore, it is possible that Claspin over-expression in different tumors may just reflect an adaptive behavior of cancer cells to promote survival in response to high levels of RS, rather than suggesting a direct role for Claspin in disease severity [116]. Indeed, there is compelling evidence, obtained in Claspin heterozygous KO mice, that Claspin generally acts as a tumor suppressor, particularly in cancer initiation [13].

Claspin over-expression not only guarantees the availability of FPC to stabilize and restart stalled replication forks and allow DNA replication and cell division, but also permits tumor cells that can activate Chk1-mediated checkpoint responses (Figure 2, bottom), providing the cell time to repair DNA damage and survive, or to escape senescence induction or apoptosis [94,109,110]. Indeed, for apoptosis to occur, Claspin must be degraded [7,8]. Over-expression of Claspin provides tumor cells with high levels of survival signals mediated by the Akt pathway [95,99].

Several studies associate Claspin expression in the tumor with increased cancer cell proliferation and tumor progression, namely migration, invasion, and metastasis formation. *CLSPN* knockdown significantly reduced the proliferative ability of renal cell carcinoma cells [95] and prostate cancer cell lines [99,100], decreased migration and invasion, and promoted apoptosis [100]. This is consistent with the requirement of Claspin degradation, namely through the action of caspases 3 and 7, for apoptosis triggering [7,8]. In addition, Claspin silencing will prevent checkpoint activation and fork stabilization in tumor cells. Therefore, tumor cells will not be able to deal with RS and DNA damage, and genome instability will increase. Finally, Claspin degradation is also required for checkpoint adaptation [47], a process through which cells can proceed to mitosis despite the presence of DNA damage. In both situations, tumor cells will end up accumulating too much damage, a feature not compatible with mitosis, which ultimately may cause tumor cells to undergo mitotic catastrophe and apoptosis. Thus, in some instances, it would be beneficial for the tumor to exploit Claspin’s functions in checkpoint activation and RS response, namely to increase survival and evade cell death (Figure 2, bottom).

Claspin has been shown to have a significant role in survival and in stress coping pathways. Claspin may be stabilized in cancer cells as a mechanism to cope with the increased instability and a stressful tumor microenvironment. For instance, EGFR, a tyrosine kinase receptor commonly upregulated and mutated in cancer, triggers an oncogenic pathway that fosters cancer cell survival and proliferation, through the activation of the PI3K/Akt and RAS–MAPK–Erk signaling pathways, respectively [117]. Claspin expression is frequently observed in EGFR-positive renal cell carcinoma cells [95], which may be due to the proliferation signaling induced through EGFR. *CLSPN* silencing was associated with decreased Erk and Akt phosphorylation [95,99], which may lead to reduced cancer cell survival. Additionally, Claspin is required for growth recovery from serum starvation by interacting with and activating the PI3K/PDK1/mTOR pathway [118]. Upon recovery from nutrient deprivation, PI3K interacts with and possibly phosphorylates Claspin, an event that promotes the recruitment of PDK1 to Claspin in a phosphorylation-dependent manner. Further phosphorylation of Claspin in its C-terminal region by PDK1 promotes the interaction of Claspin with mTOR. Claspin may act as a scaffold for the activation of the PI3K–PDK1–mTOR pathway, which, in turn, promotes amino acid biosynthesis in response to starvation, fostering cell growth [118]. This is an adaptation mechanism that may be extremely important during tumor progression, as nutrients and building blocks become scarce because of tumor growth and the activation of the immune system to try to eliminate cancer cells. The PI3K/Akt-mTOR pathway is a signaling cascade involved in several cellular processes associated with cell survival and proliferation, such as autophagy, apoptosis, angiogenesis, and chemoresistance, which are pathways frequently disturbed in cancer [119].

Recently, research has focused on *CLSPN*-associated non-coding RNA regulation and expression in cancer. The interaction and relation between the several RNA molecules that compose the transcriptome, such as non-coding RNAs, microRNAs (miRNAs), and messenger RNAs (mRNAs), form a competitive endogenous RNA (ceRNA) network, which has been shown to be important in tumorigenesis and cancer development. Salmena and colleagues [120] formulated the ceRNA hypothesis that states that non-coding RNAs, such as circular RNAs (circRNAs), directly regulate gene expression but also act as sponges, adsorbing competing miRNAs, thereby modulating their impact on the expression of target genes. For instance, an endogenous circRNA derived from exons 11–14 of *CLSPN* (hsa_circ_0011591, circCLSPN) has been implicated in glioblastoma multiforme (GBM) development. This circCLSPN forms a functional axis with miR-370-3p and USP39, which is involved in cell growth, migration, and invasion in GBM. Silencing circCLSPN resulted in inhibition of cell growth, increased apoptosis, cell cycle arrest, and reduced migration and invasion in in vitro assays. A similar effect was observed when miR-370-3p was overexpressed. USP39 and circCLSPN were both upregulated in patient biopsy samples, while miR-370-3p was downregulated. The oncogenic impact of circCLSPN is counteracted by miR-370-3p, which has pro-apoptotic, anti-proliferative, and anti-migration and invasion activities, and regulates the expression of USP39 [121].

Claspin has an important role in DNA replication both in unperturbed cells and during replication stress. It has been argued that, as tumor cells are usually under RS due to oncogene activation, the higher levels of Claspin observed in higher-grade lesions could simply reflect their higher proliferative rates as described for Ki-67 [6,88]. Indeed, Claspin has been previously suggested as a marker for aberrant proliferation, like Ki-67 [93,122]. Claspin is an integral component of the replisome, contributing to normal rates of replication [18] and assuring genome integrity by monitoring DNA replication [123]. Its expression peaks at the S-phase and whenever RS occurs, being almost absent in the remaining cell cycle [12]. Since sustained proliferation and RS are hallmarks of cancer cells, which imply active replication spots and high rates of replication, the increased levels of Claspin detected in cancer cells can simply reflect the role of Claspin in DNA replication and RS, instead of being the underlying cause of the aberrant replication per se. However, it is worth noting that the increase in Claspin expression observed in tumor samples was significantly greater than that of Ki-67, suggesting that other underlying causes may explain the increased Claspin levels [89,122]. There is also data arguing against this hypothesis [94], showing that upstream and downstream components of the ATR–Chk1 pathway are differentially regulated in primary cancer cells, and that Claspin, Tim, and Chk1 make part of a functional module that functions independently of the ATR axis. In addition, as already mentioned, Claspin and Tim seem to protect cells from endogenous RS in a way that is distinct from their ability to activate Chk1. It is believed that over-expression of these two proteins in cancer cells may allow replication fork progression and prevent genome instability in regions of the genome that are intrinsically difficult to replicate, such as common fragile sites [94]. Although spontaneous DNA repair foci are frequently seen at early steps of oncogenesis, as a consequence of oncogene-induced RS, at later stages they usually disappear as a result of the adaptation of cancer cells to RS [94]. Over-expression of Claspin and Tim is, therefore, a spontaneous event that is positively selected in early oncogenesis to protect cells from chronic RS. This is achieved through stabilization of the replisome, without triggering the ATR pathway, and provides cells with a proliferative advantage under RS, promoting replication fork progression without activating checkpoint responses, which could be detrimental to tumor growth (Figure 2, bottom) [94]. Adaptation to oncogene-induced RS mediated by over-expression of Claspin occurs at the expense of genome integrity and may therefore foster cancer progression.

Another cause for discrepancies amongst Claspin expression data can be attributed to the fact that most of the studies only focused the detection of Claspin expression on a given moment and its correlation with prognosis or other clinical parameters. The results of those studies constitute a one-moment portrait of Claspin expression, ignoring the evolutionary mutational landscape of the tumor. The correlation of Claspin expression with clinical parameters at that moment fails to fully explain Claspin’s role in the tumor pathophysiology and does not prove a causative role for Claspin in the clinical parameters studied. For instance, Jia and colleagues [98], who classified Claspin as an oncogene, found a positive association of Claspin expression with immune cell infiltration, namely, CD8^+^ T cells, macrophages, neutrophils, B cells, and dendritic cells, and with angiogenesis-associated factors, such as EGFR, and CD44 and CD133, two cancer stem cell markers [98]. Kobayashi and co-workers [97] also observed co-expression of Claspin with CD44, besides EGFR, p53, and PD-L1. Of note, one of the main distinctive features of cancer stem cells is their high proliferation rate, which is associated with high RS and, consequently (and physiologically), with increased Claspin expression and activity. In urothelial carcinoma, although Claspin expression appeared to be related to the acquisition of stem cell features (e.g., high ALDH expression), it has been shown that Claspin does not have a “driver” role in the generation of ALDH^hi^ cells, being only a “passenger” protein, probably due to its physiological functions [124]. It is possible, however, that Claspin has a role in the initial stages of cell dedifferentiation, as the silencing of Claspin in early oncogenesis may compromise transmission of the histone code to daughter tumor cells, a process crucial for epigenetic memory [11]. The inactivation of Claspin in early oncogenesis may therefore allow for dedifferentiation of tumor cells and contribute to their acquisition of stem cell features (Figure 2).

As most of the studies have only provided correlational observations, no definite conclusions can be drawn, and no hypothesis can be discarded. Thus, the possibility that Claspin over-expression in cancer can reflect oncogenic activity also has to be considered. There is evidence that genes that usually behave as tumor suppressors can sometimes acquire oncogenic activity [125,126], and this may also apply to Claspin. These “double identity” genes frequently encode proteins that are located at hubs in interaction webs and are frequently central and integrator components of different cellular mechanisms [125]. The switch from tumor suppressor to oncogenic functions is probably context-dependent and might be triggered by transcriptional or epigenetic regulation, or genetic alterations [126]. For instance, p53 is generally viewed as a tumor suppressor and is frequently silenced in cancer. p53 determines cell fate in response to stress stimuli, such as DNA damage, by directing signaling networks to appropriate cellular outcomes. Upon DNA damage, p53-checkpoint activation results in cell cycle arrest, giving the cell time to repair the damage, and triggering DNA repair pathways. After DNA is repaired, the cell cycle resumes. However, if the cell is unable to repair the damage, p53 induces cell apoptosis [127]. Thus, p53 constitutes a hub that integrates signaling cascades essential for preventing genome instability, this way maintaining cell homeostasis and preventing malignant transformation. Depending on their type and location, *TP53* mutations may result in loss or gain of function. The majority of these mutations are missense mutations located in the *TP53* DNA-binding domain and can alter the ability of p53 to bind DNA [127]. These mutations can alter p53 specificity, namely with regard to binding to DNA sequences or other proteins, which may profoundly impact p53’s transcriptional program, or may result in p53 loss of ability to bind the promoter of target genes and activate transcription. However, gain-of-function mutations can occur and confer oncogenic activity to p53, which includes, but is not limited to, stimulation of cell proliferation, metastasis formation, metabolic reprogramming, resistance to apoptosis, and genomic instability [127,128,129]. Claspin may similarly be a tumor suppressor that can act as an oncogene in specific contexts or due to specific mutations. Of note, we have identified a gain-of-function mutation in the *CLSPN* promoter [81].

### 3.2. Claspin and Response to Therapy

Cancer cells are characterized by uncontrolled proliferation and RS. To overcome the outcomes of deficient control of DNA replication, tumor cells often inactivate components of the DNA damage response (DDR), while becoming more dependent on others, particularly those involved in the maintenance of replication fork integrity. These dependencies may constitute vulnerabilities that can be exploited in cancer treatment [65]. The DDR is instrumental in maintaining genome integrity and avoiding mutagenic events that can eventually lead to cancer initiation and development. The DDR has been exploited as a source of potential therapeutic targets because the cancer cell relies heavily on its manipulation to circumvent defense mechanisms and increase genomic instability. On the one hand, breaches in this defense mechanism are frequently detected in cancer in the form of suppression of the cell cycle checkpoints. For instance, many tumors lose p53, which results in G1 checkpoint suppression, rendering the cell prone to genomic instability and consequently malignant transformation [123]. On the other hand, the DDR is often subverted by cancer cells to overcome RS and proliferate. Additionally, since the tumor abrogates certain protective pathways, it becomes more dependent on others, a feature that creates a therapeutic opportunity, namely through synthetic lethality approaches [130]. Two genes are synthetically lethal when their simultaneous inactivation results in cell death, while their individual inactivation does not. This therapeutic strategy has been perfectly illustrated with the use of Poly (ADP-ribose) protein (PARP) inhibitors in BRCA1 or BRCA2 mutated tumors. PARPs have an essential role in DDR, specifically in single-strand break (SSB) repair. This family of proteins senses DNA damage and recruits downstream effectors by catalyzing the polymerization of ADP-ribose units from NAD^+^ in target molecules. This will promote the binding of DNA breaks after SSB formation, recruitment of DNA repair effectors, and remodeling of the chromatin structure around the damaged DNA [131]. Therefore, in cancers with disrupted homologous recombination (HR), due to BRCA1 or BRCA2 mutations, the pharmacological inhibition of PARP activity results in fork collapse, genomic instability, and mitotic cell death, since the compensatory repair mechanism is absent and the cell is unable to repair the damage [131]. A similar therapeutic approach can be envisioned targeting Claspin, since, due to RS, the ATR–Claspin–Chk1 pathway is often over-activated in cancer cells [65]. In p53-deficient tumors, in particular, Claspin inhibition would further abrogate G2 and replication checkpoints. Cancer cells would not be able to arrest the cell cycle and repair DNA damage, and would eventually die in one of the following mitoses. However, to the best of our knowledge, to date, Claspin inhibitors have not been developed.

**Figure 3:**
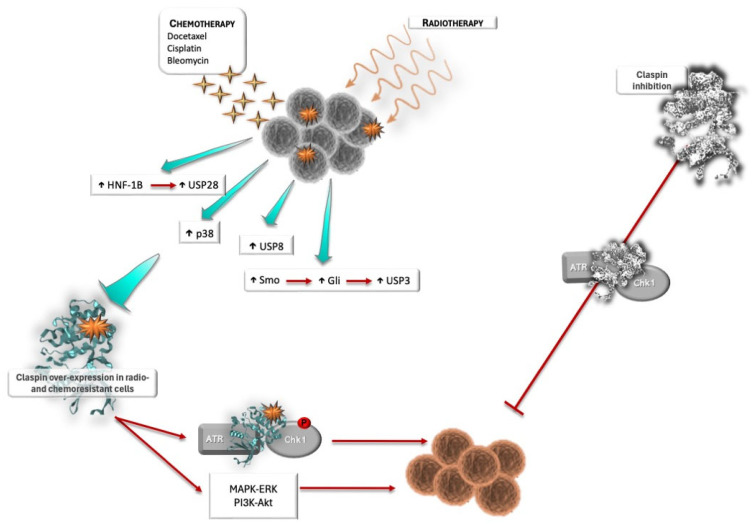
Claspin is a central player in acquired radio- and chemoresistance and cancer survival. Claspin is overexpressed in tumor cells that escape radiation- and chemotherapy, its depletion enhancing sensitivity to different cancer treatments. Claspin over-expression is generally due to the triggering of different upstream players (e.g., Smo, HNF-1β, p38) that converge on the activation of DUBs (e.g., USP3, USP8, USP28) and Claspin stabilization, thereby allowing Claspin-dependent ATR–Chk1 signaling activation and cell cycle arrest, providing the cell time to repair DNA damage and survive. Claspin over-expression also allows survival of cells exposed to genotoxics through activation of survival pathways, as those mediated by Akt and Erk. Therefore, Claspin inhibition may constitute a therapeutic strategy for chemo- and radio-sensitization.

Mounting evidence places Claspin as a central component in acquired radio- [132,133] and chemoresistance [69,99,119,134] and cancer survival (Figure 3). Therefore, therapeutic Claspin inhibition is increasingly gaining relevance. Radiation- and chemotherapy induce DNA damage to an already genome-unstable cancer cell, increasing RS and promoting cell death. Cancer cells rely on the activation of survival mechanisms, such as the DDR, usually through the abnormal expression of proteins implied in those mechanisms, to cope with DNA damage [135]. Choi and colleagues [132] found that Claspin was overexpressed in lung cancer brain metastasis that escaped radiotherapy. The authors observed that Claspin depletion enhanced radiation sensitivity in vitro in radioresistant lung cancer cells, but also in vivo using xenograft models of lung cancer brain metastasis. Interestingly, the increased cell death was more dramatic at higher radiation doses (e.g., 4Gy) than at the lower doses used in conventional radiotherapy (around 2Gy or less). The median survival time post radiation therapy was also increased [132]. Glioblastoma, an extremely aggressive tumor, also presents high levels of radio-resistance following the initial rounds of treatment. Smoothened (Smo), a component of the Hedgehog signaling pathway that triggers the activation of transcription factors collectively known as Gli, is consistently overexpressed following radiation therapy and is associated with radio-resistance [133]. Smo-mediated radio-resistance is achieved through USP3 transcription activation, which reduces Claspin polyubiquitination and, consequently, its proteasomal degradation. Therefore, Smo expression promotes Claspin stabilization and thus Claspin-dependent ATR–Chk1 checkpoint activation and cell cycle arrest, providing the cell time to repair DNA damage, which results in radio-resistance (Figure 3) [133]. Radiation causes double-strand breaks (DSBs), which are the most deleterious type of DNA damage to the cell. DSBs are repaired either through non-homologous end joining (NHEJ) or HR, depending on the complexity of the lesion and the cell cycle phase. HR can be activated via the ATR–Chk1 pathway. Therefore, if Claspin is overexpressed in radioresistant cells through the deubiquitination action of USP3, the cancer cells can then rely on the activated ATR–Chk1 pathway to overcome the radiation-inflicted damage, either by the repair of damage or by another genome-protective ATR–Chk1-associated action, such as cell cycle arrest and replication fork stability [135]. Smo inhibition sensitizes cancer cells to radiation, as in this situation, USP3 is not expressed, and Claspin is polyubiquitylated and degraded in the proteasome [133]. Therefore, there is no ATR/Chk1 checkpoint activation, and cells are not able to repair DNA damage, which promotes a G2 arrest (as the ATM/Chk2 pathway is still intact) and redistribution of cells to more radiosensitive phases of the cell cycle, thus radiosensitizing cancer cells and promoting apoptosis [133]. Progression into mitosis with extensive accumulated DNA damage also leads to radio-sensitization, as irradiated cells end up dying in one of the following mitoses by mitotic catastrophe.

Cancer therapy using cytotoxic agents is widely used in the clinic. Claspin expression was also associated with resistance to chemotherapy in several cancers (Figure 3). Docetaxel is an antineoplastic agent that promotes the stabilization of microtubules by inhibiting depolymerization, which results in mitotic cell cycle arrest and consequent cell death [136]. Claspin expression was found to be enhanced in docetaxel-resistant prostate cancer cells, and functional studies demonstrated that docetaxel sensitivity could be restored by silencing Claspin, which was associated with reduced activation of the Akt and Erk pathway, and a consequent decrease in survival signals [99]. Therefore, Claspin deserves consideration as a target in the treatment of docetaxel-resistant tumors. In addition, Claspin has been reported as a source of resistance to other anti-cancer agents, such as platinum salts (e.g., cisplatin) and bleomycin. In ovarian clear cell carcinoma and endometriosis, high levels of HNF-1β induce G2 cell cycle arrest and survival of cancer cells through Chk1 activation, following genotoxic stress induced by bleomycin, a cytotoxic antibiotic [134]. The survival mechanism relies on Claspin stabilization through a post-transcriptional mechanism (Figure 3). Upon genotoxic stress, HNF-1β prevents Claspin ubiquitin-mediated proteasomal degradation through the action of USP28, therefore promoting Claspin stabilization, and inducing Chk1 activation and G2 cell cycle arrest, which provides cancer cells more time to repair DNA damage and survive [134]. High-grade serous ovarian carcinoma frequently develops resistance to platinum-based therapies, namely cisplatin [137]. Cisplatin is a cytotoxic drug that exerts its effect by interacting with DNA, forming DNA adducts. These adducts can form mono, inter, or intra-strand DNA crosslinks that block the cell cycle at S, G1, or G2-M phases, ultimately leading to apoptosis [138]. The observed resistance seems to be due to over-expression of USP8, which suppresses apoptosis induction, due to stabilization of Claspin (Figure 3). Silencing of USP8 reverts resistance to platinum salts through activation of caspase-7 and Claspin degradation, thereby promoting apoptosis [137]. Claspin phosphorylation by p38 was also shown to promote cell survival after treatment with cisplatin [69]. p38 is activated and phosphorylates Claspin, resulting in a delay in S-phase progression to give the cell time to cope with DNA damage (Figure 3). This way, the p38–Claspin pathway protects cells from DNA damage occurring during the S-phase [69], representing another resistance mechanism to cisplatin and, therefore, a potentially targetable pathway for cancer treatment [69]. Over-expression of Claspin also seems to underlie acquired resistance to cisplatin in urothelial carcinoma [120]. In summary, Claspin over-expression in tumors and, particularly, in therapy-resistant tumors, appears to be an adaptation mechanism of cancer cells to cope with the stress imposed by therapy. However, this might also represent a therapeutic opportunity to enhance treatment success in resistant tumors. In addition, Claspin over-expression due to stabilization can be used as a therapy-selection marker in tumors resistant to chemotherapy agents, namely platinum-based salts [134]. In these tumors, Claspin stabilization may promote tumor cell survival through activation of Chk1-mediated G2 checkpoint responses and cell cycle arrest. Of note, G2-cell synchronization radiosensitizes cells. Therefore, analysis of Claspin expression in chemotherapy-resistant tumors may allow the selection of patients who would benefit from radiation therapy.

Although in all the above-described situations Claspin over-expression was not the direct cause of resistance to therapy but resulted from the upstream aberrant activation of proteins that are able to interfere with Claspin’s half-life, interfering with Claspin expression determined cell fate in all these settings. Therefore, Claspin may constitute an attractive therapeutic target for chemo- and radio-sensitization [43].

In triple-negative breast cancer, increased GSK3-β expression is associated with a worse prognosis. GSK3-β activation also interferes with Claspin stabilization [139]. GSK3-β is involved in the regulation of protein synthesis, cell proliferation, cell differentiation, neuronal signaling, immune function, and inflammation, and has been found to be upregulated in several cancers, including breast cancer [139]. GSK3-β inhibitors were found to suppress the growth of breast cancer cells and to sensitize breast cancer cells to irinotecan, a chemotherapeutic agent [140]. GSK3-β interacts directly with Claspin, modulating Claspin–SCFβTrCP interaction, targeting Claspin for polyubiquitination and proteasomal degradation. Inhibition of GSK3-β enhances Claspin half-life and, hence, Chk1 activation and pro-survival mechanisms [141]. Combined inhibition of GSK3-β and Chk1 effectively inhibited cancer proliferation, both in 2D- and 3D-models, suggesting that this can be an effective strategy to inhibit cancer cell growth and survival [141]. Claspin also seems to play a role in the response to camptothecin, leading to Chk1 activation upon association with BRCA1, this way triggering the DDR [46]. Therefore, targeting Claspin may be a useful strategy either on its own or in the context of synthetic lethality [43], as tested with simultaneous targeting of GSK3-β and Chk1 [141].

Although Claspin is an essential gene for the maintenance of cell viability and homeostasis, the use of Claspin inhibitors may still be viable and achievable without too much (or acceptable) toxicity to healthy cells due to the intrinsic features of cancer cells that are absent in healthy ones [65]. For instance, while differentiated healthy cells lose the ability to proliferate, tumor cells divide uncontrollably and are in constant RS, and therefore are a lot more dependent on the RS response. In addition, in the presence of DNA damage, healthy cells have a myriad of mechanisms and pathways to overcome the problem and survive. These include proper functioning of cell cycle checkpoints and intact DNA repair pathways. In contrast, in tumor cells, several of these pathways are usually inactivated. Although these deficiencies are used by tumors to bypass cells’ defenses, they also constitute Achilles’ heels that may be therapeutically exploited, namely by hitting still functional pathways. Consequently, tumor cells will end up accumulating too much damage and end up dying in one of the following mitoses. These differences between healthy and cancer cells may provide a therapeutic window for the use of Claspin inhibitors.

Although much research is still needed, Claspin is now viewed as an attractive therapeutic target, and much research is ongoing to validate it as such. Further research is needed in order to translate this knowledge into therapeutic tools. However, as Claspin biological behavior is uncovered, Claspin-centered therapies appear to become a gradually closer reality.

## 4. Final Thoughts and Perspectives

There are many routes to cancer. During oncogenesis, tumor cells accumulate a series of changes (genetic, epigenetic, post-transcriptional, and post-translational) that ultimately result in the acquisition of a set of “new” biologic abilities: sustained proliferation, resistance to growth inhibitors, apoptosis resistance and increased survival, immortality, angiogenesis, invasion, and metastasis formation. Different tumors acquire given abilities at different timings during oncogenesis, and the order of ability acquisition is also variable between tumors. A well-established enabling feature that promotes tumor development is genome instability, which is crucial for the inactivation of tumor suppressor genes and the activation of oncogenes. Genome instability is particularly important in the early stages of oncogenesis. Healthy cells have developed several mechanisms to maintain genome integrity, namely monitoring of DNA replication and quality, and the presence of replication errors, and through the existence of checkpoints during the cell cycle, when the quality of DNA is checked before and after replication, and when the cell verifies if DNA replication has been completed. When problems are found, these checkpoints are activated and the cell cycle arrested, so that the cell has time to attempt to repair DNA or deal with other problems through localized responses. If the damage is too extensive or irreparable, the cell is then induced to die through apoptosis. Successful tumor cells must overcome these genome protection barriers.

**Figure 4:**
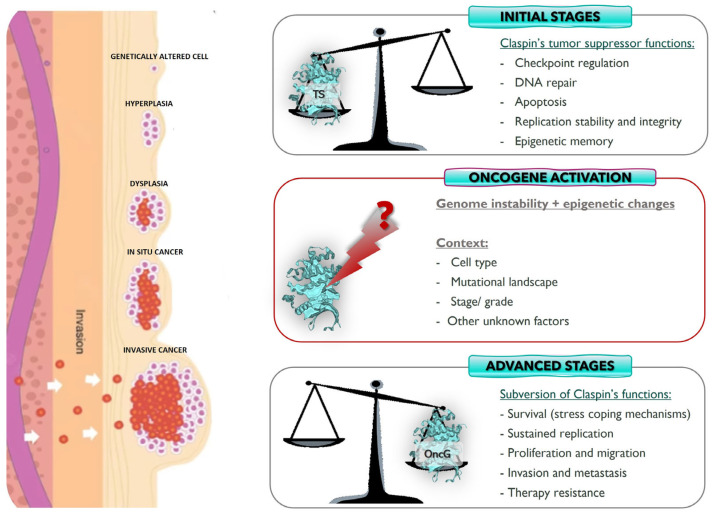
Proposed model for Claspin’s role in cancer. At pre-malignant and initial stages of cancer development, Claspin acts as a tumor suppressor, preventing malignant transformation. This is achieved by Claspin’s tumor suppressor functions that include its role in DNA damage and replication checkpoint activation, replication protection, DNA repair, apoptosis, and epigenetic memory. CLSPN’s inactivation at such early stages is known to promote carcinogenesis. However, cancer is an evolving entity. As oncogenes are activated, cancer cells start feeling the pressure of replication stress (RS) and must adapt. Adaptation seems to involve exploitation of Claspin’s functions in the RS response, through its role in the replisome and FPC, which is independent of the ATR–Chk1 checkpoint activation, but also through its role in Chk1-mediated checkpoint responses. However, there are many routes to cancer, and different cancer cells may adopt distinct strategies to survive and proliferate. The strategies adopted may depend on the cell type and context, the mutational landscape and the stage of carcinogenesis. Through its stabilization and expression, Claspin promotes survival by different pathways, specifically, through stress coping mechanisms. For instance, Claspin-sustained expression may inhibit apoptosis, promoting cancer cell survival, namely through survival signals mediated by Akt. Claspin is also involved in mechanisms that allow the uncontrolled replication of tumor cells (RS), and possibly also contributes to tumor migration, invasion, and metastasis formation. Importantly, Claspin over-expression seems to underly several pathways implicated in therapy resistance, which turns Claspin targeting, in single or combined therapeutic regimens, into an attractive possibility.

Figure 4 summarizes our proposed model for the role of Claspin in cancer. Claspin is involved in many of the mechanisms that guarantee genome integrity. It is part of the replication fork protection complex (FPC) and the replisome, monitors DNA replication, and is involved in checkpoint activation, as well as the activation of different DNA repair pathways [6] and of other survival mechanisms, such as those mediated by EGFR and mTOR [117,118]. Claspin should be considered a tumor suppressor, as its inactivation should facilitate cancer cell development through genome instability and the accumulation of genetic changes. Indeed, this was clearly demonstrated in studies of oncovirus infections (e.g., such as HR-HPV). For cervical cancer development, HR-HPV must inactivate the Chk1-mediated checkpoint through Claspin degradation, in addition to the inactivation of pRb and p53 [71]. We have shown that a *CLSPN* mutation associated with breast cancer (c.1574A>G; p.Asn525Ser) resulted in either Claspin degradation or decreased Chk1 phosphorylation and, as such, in deficient checkpoint activation [81]. Claspin inactivation, in the early stages of carcinogenesis, may also abolish an evolutionary conserved genome defense mechanism, used to deal with RS, that is triggered by activation of p38. Claspin activation by p38 allows cells to revert oncogene-induced RS in an ATR–Chk1-independent manner, and thereby decreases the overall burden of DNA damage and protects the cells from genome instability [70]. An additional mechanism that guarantees genome integrity involves the activation of NF-kB in the presence of oncogene-induced RS. NF-kB can act as a tumor suppressor and prevent cancer development through the activation of the Claspin–Chk1-mediated checkpoint response, which, early in carcinogenesis, will prevent the accumulation of genetic mutations [80]. In addition, Claspin inactivation may allow cells with extensive DNA damage to undergo checkpoint adaptation and survive despite the damage, therefore being able to proceed through the cell cycle and divide. Furthermore, Claspin seems to be a key protein in epigenetic memory, guaranteeing that cell lineage features are maintained in the progeny. Its inactivation may interfere with the transmission of chromosome structural information onto daughter tumor cells and allow cell dedifferentiation [11]. Therefore, Claspin inactivation may facilitate oncogenesis in several ways.

However, as tumors accumulate changes and oncogenes are activated, in more advanced stages of oncogenesis, Claspin-mediated pathways may conversely promote tumor progression, as they may help to foster tumor cell addiction to checkpoint kinase signaling, which is required for the prevention of further genome instability and for promoting cancer cell survival. In line with this, it has been observed that oncogene activation (e.g., EGFR, HER2, PI3K/Akt) promotes Claspin stabilization and expression [95,99,117]. This sustained Claspin expression can also be important for the survival of cancer cells, as Claspin degradation is required for apoptosis induction when the DNA damage is too extensive, as is usually the case in cancer cells. Indeed, acquisition of resistance to apoptosis induction (for instance, through p53 inactivation) is a key hallmark of cancer. Although Claspin expression can be exploited by cancer cells for their survival, Claspin’s role as an oncogene should be regarded with caution since, generally, its expression and activation only seem to reflect and be secondary to triggering of oncogene-induced pathways, to the ongoing RS, and to the acquisition of stemness features (e.g., CD44, CD133, and ALDH expression) [97,98]. As described above, Claspin is an integral component of the replisome and FPC, whose expression peaks in situations of RS. As tumors become out of control and their stemness features increase (e.g., undifferentiation, and high proliferation and replication rates), Claspin expression increases, which suggests that tumor cells exploit the physiological role of Claspin in response to RS and in the FPC. Nevertheless, we have described a *CLSPN* promoter mutation (c.−68C>T) that seems to be a gain-of-function mutation [81], a feature that usually characterizes oncogenes. Therefore, this matter deserves further study.

As described and discussed above, there are data correlating Claspin expression with both better and worse prognosis, and other clinical parameters, in different types of cancer. As already highlighted, different tumors follow different routes to cancer. Their mutational landscape will be different, and the sequence of events and ability acquisition of tumor cells will also be distinct. In addition, distinct tumors become addicted to different oncogene-induced pathways and will inactivate distinct tumor suppressor genes and respective tumor suppressive mechanisms. These differences may explain the apparently opposing correlational findings regarding Claspin expression and clinical outcomes. This was clearly demonstrated for breast cancer, in which low Claspin expression was correlated with a worse overall survival but only when tumor cells concomitantly presented p53 inactivation (apoptosis resistance) or HER2 over-expression (increased proliferation and survival signals; resistance to apoptosis induction), the opposite being observed in cells with wt *TP53* (which can still be induced to die by apoptosis) or low-to-negative expression of HER2 (lower survival signals; higher sensitivity to apoptosis induction) [80]. Distinct correlational findings may go beyond Claspin over-expression and reflect other changes present in tumor cells, namely at the level of apoptotic and DNA repair pathways, which will determine the fate of tumor cells and tumor cell evolution and adaptation to the selective pressures of the tumor’s microenvironment. Bianco and colleagues [94] have shown that different clones of cancer cells acquired different properties and adopted distinct strategies to cope with RS, some of which could not be fully explained by over-expression of Claspin and Tim. A more detailed analysis of the mutational (and other events—epigenetic, post-transcriptional, and post-translational) landscape will probably clarify the apparent contradictory correlational observations.

There are also important data regarding the role of Claspin in the response to treatment. Generally, it was observed that several pathways involved in tumor resistance to either chemo- or radiation therapy involved downstream stabilization and sustained expression of Claspin [69,99,124,132,133,134]. Even though this increased expression of Claspin is secondary to the activation of pathways mediated by different proteins (e.g., HNF-1ß, Smo, p38), it highlights the possibility of exploiting Claspin as a therapeutic or sensitization target, using approaches that promote its inactivation and/or degradation. Although, to date, the majority of efforts to enhance responses to chemo- and radiation therapy have mainly focused on “classical” players of checkpoint responses (e.g., ATR, Chk1) and DNA damage repair (e.g., PARPs), scenarios can be envisaged using Claspin [43], either as the sole target, or in the context of synthetic lethality approaches (e.g., in cells presenting *TP53* mutations).

## 5. Conclusions

In conclusion, current data clearly highlight a role for Claspin in cancer. In early stages, Claspin activation may counteract tumor development, constituting an important barrier that cancer cells must overcome. However, as oncogenesis progresses and replication stress (RS) increases, cancer cells may start to exploit the RS response, in which Claspin plays a pivotal role. Being a hub onto which several therapy-resistance pathways converge, Claspin constitutes an attractive target for radio- and chemo-sensitization.

## Abbreviations

The following abbreviations are used in this manuscript:

AKT	AK strain transforming
ALDH	Aldehyde dehydrogenase
APC	Anaphase promoting complex
ATM	Ataxia telangiectasia mutated
ATR	Ataxia telangiectasia and Rad3-related protein
ATRIP	ATR interacting protein
BARD1	BRCA1-Associated RING Domain 1
BRCA1	Breast Cancer gene 1
Bub1	Budding uninhibited by benzimidazoles 1
Cdc	Cell division cycle
Cdh1	Cdc20 homolog 1
CDKs	Cyclin-dependent kinases
Chk1/2	Checkpoint kinase 1/2
CK1γ1	Casein kinase 1 gamma 1
DDB	Damaged DNA binding protein
EGFR	Epidermal Growth Factor Receptor
ERK	Extracellular signal-regulated kinase
ETAA1	Ewing’s tumor-associated antigen 1
FANC	Fanconi
FANCD2	Fanconi Anemia Complementation Group D2
GSK3-β	Glycogen Synthase Kinase 3-beta
HER2	Human Epidermal Growth Factor Receptor 2
HNF-1β	hepatocyte nuclear factor 1-beta
Ki67	Antigen Kiel 67
MAPK	Mitogen-activated protein kinases
MCM	Mini-chromosome maintenance
MSH6	MutS homolog 6
mTOR	Mammalian target of Rapamycin
OZF	Only Zinc-Finger
PCNA	Proliferating cell nuclear antigen
PDK1	Phosphoinositide-dependent kinase 1
PD-L1	Programmed death ligand 1
PI3K	Phosphoinositide 3-kinase
PMS2	Postmeiotic segregation increased 2
Pol/POL	Polymerases
pRb	Retinoblastoma protein
RAD51	DNA repair protein
Ras	Rat sarcoma oncogene
RFC	Replication Factor C
RPA	Replication protein A
SCFβTrCP	Skp1-Cullin-F-box-protein ubiquitin ligase complex
SLF2	SMC5-SMC6 Complex Localization Factor 2
STAT3	Signal transducer and activator of transcription 3
Tim	Timeless
TOPBP1	Topoisomerase II binding protein 1
TRIM21	Tripartite motif protein 21
USP	Ubiquitin-specific proteases
3′-UTR	3′-untranslated region
XPC	Xeroderma pigmentosum protein C
